# Dual-energy computed tomography collagen density mapping of the temporomandibular disc

**DOI:** 10.1186/s12880-025-01935-3

**Published:** 2025-09-15

**Authors:** Sevtap Tugce Ulas, Thomas Matthias Wittig, Virginie Kreutzinger, Friedemann Göhler, Erin C. Argentieri, Torsten Diekhoff, Katharina Ziegeler

**Affiliations:** 1 Department of Radiology, Charité – Universitätsmedizin Berlin, Humboldt – Universität zu Berlin, Freie Universität Berlin, Campus Mitte, Berlin, Germany; 2 Department of Radiology and Biomedical Imaging, University of California - San Francisco, San Francisco, USA; 3 Department of Radiology, Immanuel Clinic Ruedersdorf, Brandenburg Medical School, Seebad, Ruedersdorf, Germany

**Keywords:** Dual-Energy CT, Collagen, Temporomandibular joint

## Abstract

**Background:**

The aim of this study was to evaluate the feasibility of dual-energy computed tomography (DECT)-derived collagen density mapping of the temporomandibular disc and to analyze the association with CT-detected joint degeneration and clinically relevant covariates such as age and sex.

**Methods:**

A total of 155 patients who underwent DECT scan (135 and 80 kVp with 180 and 500 mA) of the neck with clinical indication unrelated to temporomandibular joint (TMJ) disease were analyzed. Collagen density maps were calculated. Region of interest analysis was performed to assess collagen densities of the temporomandibular discs. Osteoarthritis of the TMJ was assessed on a numeric scale of 0 to 2. Generalized estimating equations (GEE) were used to assess the association of discal collagen density (DCD) and patient specific factors.

**Results:**

Osteoarthritis of the TMJ was observed in 86 joints (27.2%), of which 16 (4.7%) exhibited severe degeneration. Unadjusted mean collagen density of the temporomandibular disc was 142.3 HU (SD 42.7; range 11.4–243.2). GEE in the whole cohort showed a significant negative association between DCD and presence of any degeneration (beta − 11.4, 95%CI -22.3, -0.6, *p* = 0.039) and female sex (beta − 22.8, 95%CI -36.7, -8.8, *p* = 0.001). Thus, when adjusting for degeneration and age, adjusted mean DCD was 124.4 HU (SD 40.7) in females and 146.3 HU (SD 42.1) in males (*p* < 0.001).

**Conclusions:**

Females with TMJ degeneration tended to show lower DCD values compared to males. The quantification of collagen density in the disc provides promising complementary information on structural and functional integrity of the TMJ.

## Background

The temporomandibular joint (TMJ) is characterized by its complex anatomical structure [1]. It connects the mandible to the temporal bone and enables a variety of movements, including rotation and translation, which are essential for fundamental functions such as chewing and speaking [2]. Due to its intricate structure and load-bearing function, the TMJ is subject to degenerative changes, which have been associated with temporomandibular disorders and related morbidity. In recent years, dual-energy computed tomography (DECT) has emerged as a promising imaging technique for non-invasive characterization of tissue composition. By utilizing low- and high-energy spectra, DECT enables material-specific attenuation analysis [3], as successfully demonstrated for ligamentous structures [4, 5] and intervertebral discs [6, 7]. Its potential for evaluating the biomechanical properties of the TMJ remains to be explored. Since collagen plays a crucial role in maintaining the structural integrity and function of the articular disc, changes in its density may thus indicate altered biomechanical properties and serve as early indicators of joint degeneration, potentially before clinical symptoms manifest, enabling early intervention. Furthermore, previous studies have indicated that females generally exhibit lower collagen density in various tissues [8].

The TMJ is formed by the head of the mandible, which articulates within the glenoid fossa of the temporal bone [9]. The articular disc serves as a crucial connection between these two structures, playing a significant role in the mobility of the TMJ. This disc consists of a biconcave-shaped fibrocartilaginous structure that divides the joint into two compartments and functions as a shock absorber [10, 11]. Collagen is a major component of the articular disc, critical for maintaining structural integrity and contributing to stability and elasticity within the TMJ [12]. This characteristic enables the articular disc to adapt to the shape of the joint surfaces and absorb pressure during chewing activities [13]. With increasing age, malalignment or overuse, degenerative changes may occur in the TMJ. TMJ disorders, also referred to as temporomandibular dysfunction, are a multifactorial condition that affects 5–12% of the population and can lead to debilitating pain [14, 15]. Degenerative processes in the TMJ have been associated with alterations in collagen structure, potentially reducing tissue resilience and contributing to clinical symptoms such as pain and restricted mobility [14].

The objective of this proof-of-concept study was to assess the collagen density of the articular disc in a cohort without inflammatory disease using DECT-driven collagen density mapping and to investigate clinical covariates such as age, sex, and TMJ degeneration.

## Methods

### Subjects

In this retrospective study we included all patients who underwent a DECT scan of the neck with clinical indication between May 2019 and August 2020 in the Department of Radiology of our university hospital. Imaging indications and associated known conditions of the patients included the following: head and neck cancers, with or without previous radiation therapy (such as laryngeal cancer, oropharyngeal cancer, and hypopharyngeal cancer), inflammatory neck diseases (including acute tonsillitis, cervical phlegmon, and postoperative imaging following tonsillectomy), as well as other conditions (such as various tumor types including thyroid carcinoma, esophageal carcinoma, malignant melanoma, and cervical lymphadenopathy of unknown origin). Patients with known rheumatic joint diseases or hyperparathyroidism, as well as those with insufficient image quality for the application of further reconstruction algorithms were excluded.

The study was approved by the local ethics committee (EA1/247/21). All patients gave their written informed consent to scientific use of their imaging and clinical data prior to the DECT scan, which is standard by protocol in our institution. All procedures performed in studies involving human participants were in accordance with the ethical standards of the institutional and/or national research committee and with the 1964 Helsinki Declaration and its later amendments or comparable ethical standards.

### Imaging technique

The DECT scan of the neck was performed using a 320-row single-source CT scanner (Canon Aquilion ONE Vision, Canon Medical Systems) utilizing the wide-volume mode. This mode allows for non-helical volume imaging with a z-axis coverage of up to 16 cm, enabling complete visualization of the TMJ region in a single acquisition per energy level. Dual-energy imaging was achieved through two sequential volume scans at low and high tube voltages (80 kVp and 135 kVp, with 500 mA and 180 mA, respectively). The rotation time per scan was 0.5 s, and the change-over time between the two acquisitions—i.e., the time required to switch from one energy level to the other—was also 0.5 s, resulting in a total acquisition time of approximately 1 s. Each energy level was acquired with one full rotation. A body-weight-adapted split-bolus injection of contrast media (Ultravist 370, Bayer) was administered prior to scanning. Image reconstruction was performed with 0.5 mm slice thickness using a medium soft tissue kernel (without beam hardening correction) and a 0.5 mm bone kernel in coronal, transverse, and sagittal planes.

### DECT image processing and region of interest analysis

Collagen density maps were generated using a vendor-specific software (Dual-Energy Raw Data Analysis, Version 6, Canon Medical Systems) directly on the CT console. A dual-energy gradient of 1.1 was applied within the three-material decomposition algorithm, optimized for collagen differentiation [16, 17]. This parameter enhances sensitivity to the specific attenuation characteristics of collagen across the two energy spectra. The collagen maps were reconstructed with a primary slice thickness of 0.5 mm. All images were pseudonymized.

**Fig. 1:**
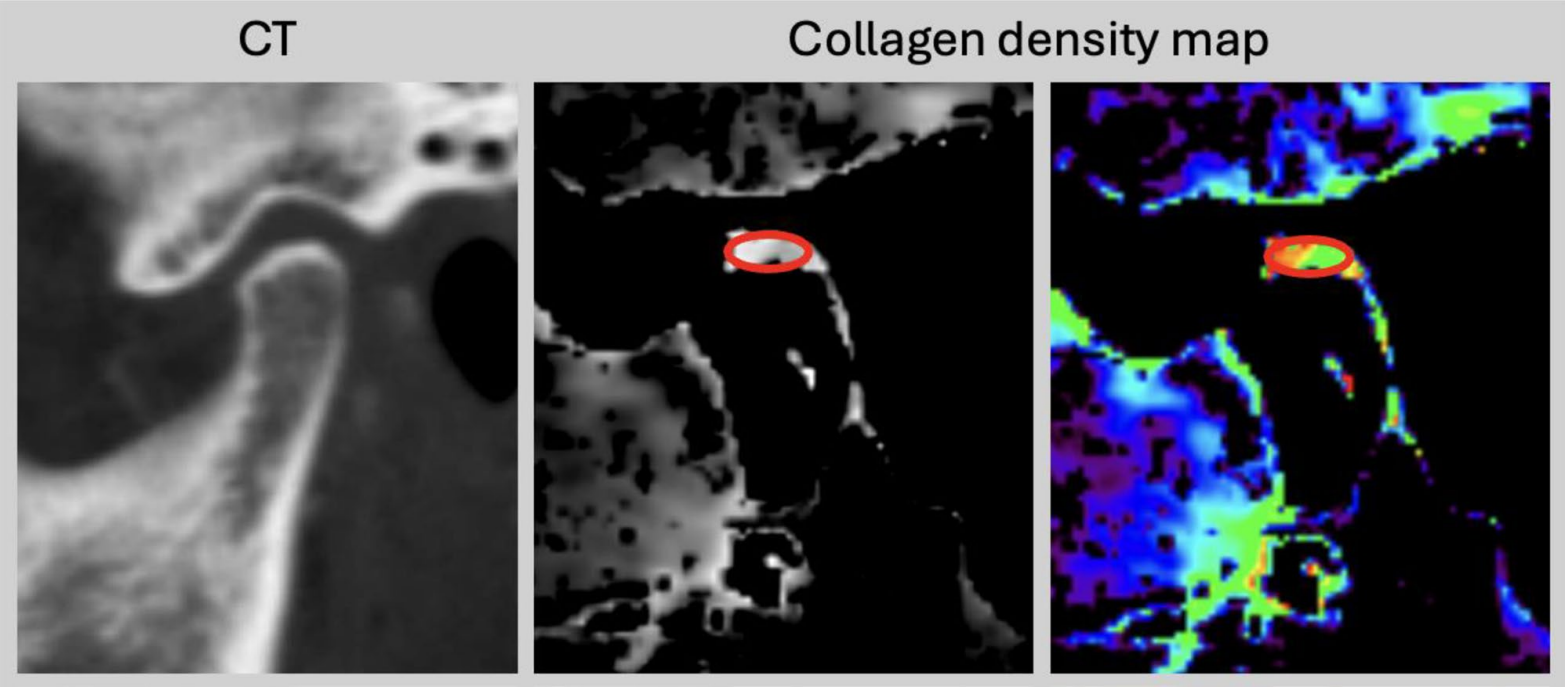
Quantitative measurement. Example image of clinical CT and collagen map with region of interest placement (red oval) in the temporomandibular joint

A standardized region of interest (ROI) analysis was performed by a specially trained research student (T.M.W.) using the collagen density maps (Fig. 1). All measurements were conducted on the sagittal orientation using a standardized elongated oval-shaped ROI with a size of 8.0 mm^2^ in the TMJ for both sides separately with a dedicated software (Horos v.2.2.0, The Horos Project). ROI placement was performed according to predefined anatomical landmarks, specially at the widest convexity of the mandibular condyle, to ensure comparability across patients. Care was taken to position the ROIs strictly within the articular space, explicitly avoiding adjacent structures such as tendons or soft tissue. In cases of uncertainty, ROI placement was re-evaluated in consensus with a senior radiologist. Mean densities (Hounsfield unit, HU) and standard deviations (SDs) were collected.

### Image reading

**Fig. 2:**
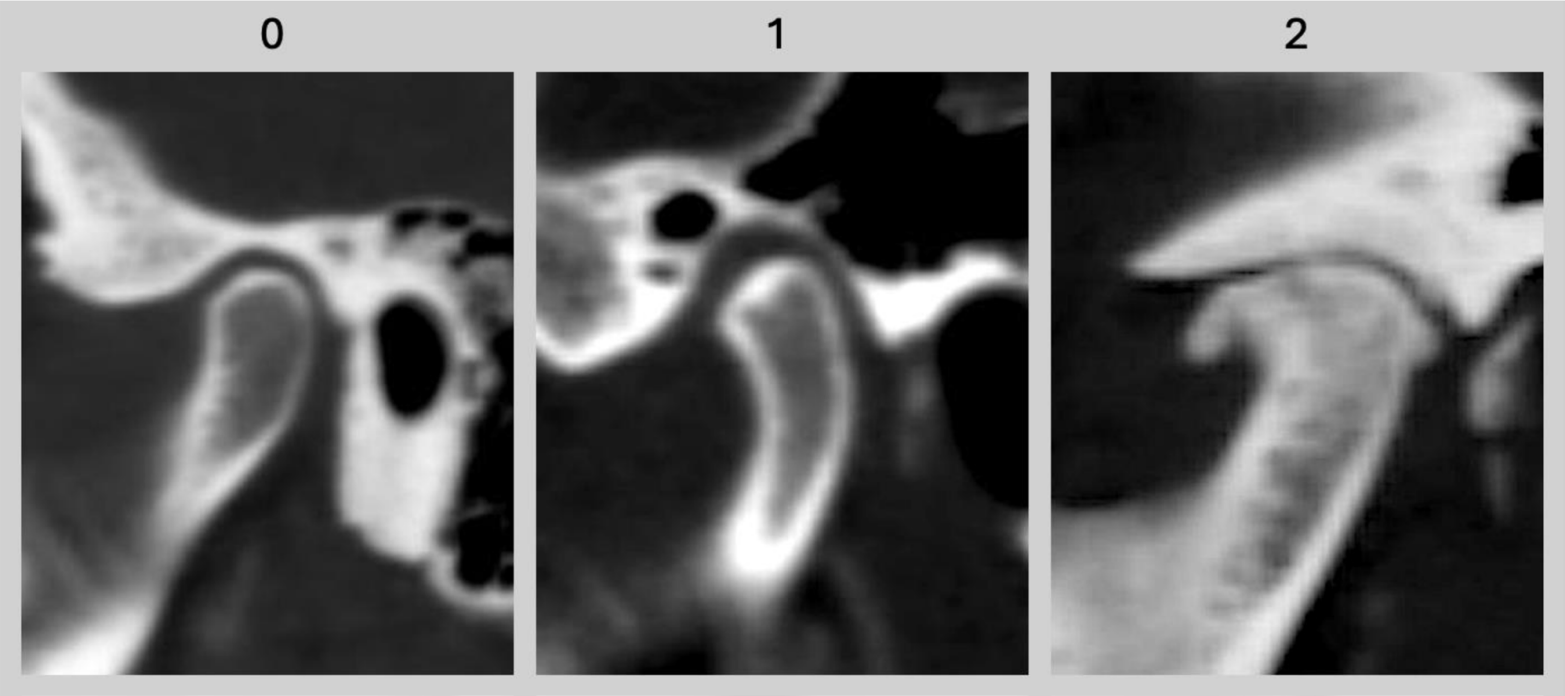
Temporomandibular joint osteoarthritis. **0**: Normal relative size of the condylar head; and no subcortical sclerosis or articular surface flattening; and no deformation due to subcortical cyst, surface erosion, osteophyte or generalized sclerosis. **1**: Normal relative size of the condylar head; and subcortical sclerosis with/without articular surface flattening; or articular surface flattening with/without subcortical sclerosis; and no deformation due to subcortical cyst, surface erosion, osteophyte or generalized sclerosis. **2**: Deformation due to subcortical cyst, surface erosion, osteophyte or generalized sclerosis

The 135 kVp CT bone kernel reconstructions were assessed by a senior radiologist (K.Z.) with 8 years of experience in musculoskeletal imaging for TMJ osteoarthritis using a 3-point numeric scale (0 = normal, 1 = moderate, 2 = severe). The grading was based on characteristic radiological features: normal joints (grade 0) showed no structural abnormalities; moderate degeneration (grade 1) included joint space narrowing and small osteophytes; severe degeneration (grade 2) was defined by the presence of extensive osteophytes, subchondral sclerosis, and significant joint deformity (see imaging examples in Fig. 2). A second radiologist (S.T.U.) with 6 years of experience in musculoskeletal radiology independently graded TMJ degeneration to assess inter-rater reliability. The readers were blinded to all clinical data.

### Statistical analysis

Statistical analysis was performed using Python Version 3.11.8. Generalized estimating equations (GEE) were used to assess the association of discal collagen density and patient specific factors, treating each TMJ as a separate unit of observation, but accounting for lack of independence between both measurements within one individual. Discal collagen density was defined as the outcome marker and TMJ degeneration, age, sex and prior radiation therapy were defined as predictors. After assessing influence of each of these factors, adjusted mean collagen densities were calculated and compared between subgroups using linear models. Due to the explorative nature of this study, we refrained from correcting for multiple comparisons and considered a p-value < 0.05 statistically significant.

## Results

### Subjects

In total 178 patients were evaluated for inclusion. Three patients were excluded because of known rheumatological diseases, and six patients were excluded because of insufficient image quality, and 14 patients had received two exams, only the first of which was used in this analysis. Thus, 155 patients (125 male, 30 female, 310 total TMJs) with a mean age of 64.6 (SD 11.9; range 28–88 years) were included in the analysis. There was no significant difference in age between males and females (mean age 65.9 vs. 62.8; *p* = 0.388). DECT imaging was performed for staging of malignant disease in the majority of patients (96.8%; 150/155), and for evaluation of inflammatory conditions (e.g. abscess) in a small minority (3.2%; 5/155).

### Degeneration of the TMJ

**Fig. 3:**
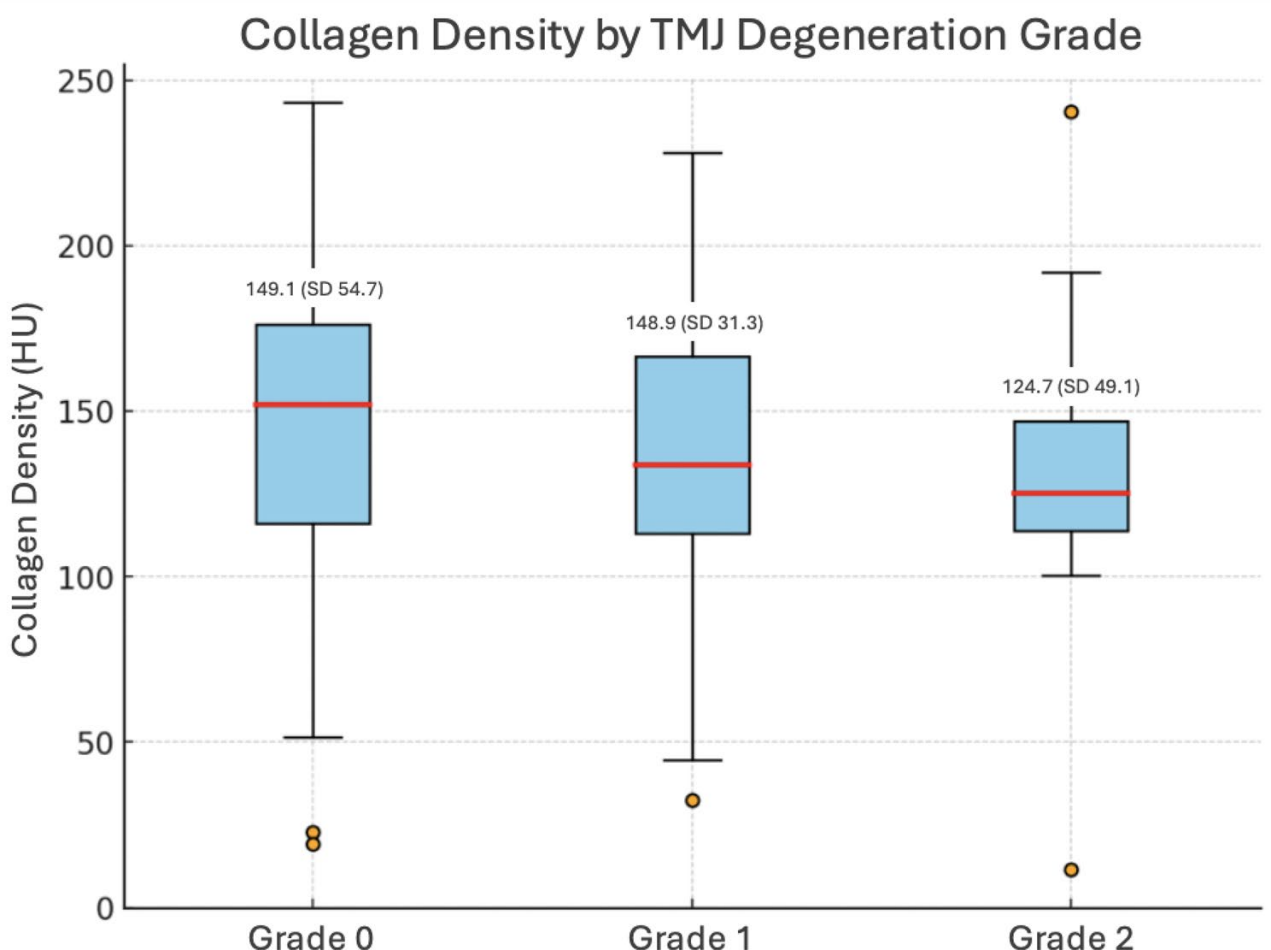
Boxplots of discal collagen density by temporomandibular joint degeneration grade. The mean discal collagen density was 149.1 (SD 54.7) in normal TMJ (grade 0), 148.9 (SD 31.3) in moderate TMJ osteoarthritis (grade 1) and 124.7 (SD 49.1) in severe TMJ osteoarthritis (grade 2)

As detailed above, 310 TMJs from 155 patients were available for analysis. Using our simplified grading system, degeneration of the TMJ was observed in 87 TMJs (27.2%), of which 16 (4.7%) exhibited severe degeneration – a boxplot of collagen densities compared with TMJ degeneration is given as Fig. 3. An unadjusted comparison of different grades of degeneration between males and females did not yield statistically significant results (*p* = 0.066): no, moderate and severe degeneration, respectively, were found in 74.4% (186/250), 21.6% (54/250) and 4.0% (10/250) of male TMJs, and 61.7% (37/60), 28.3% (17/60) and 10.0% (6/60) of female TMJs. Degeneration was equally common on both sides with 24.5% (38/155) on the right vs. 31.6% (49/155) on the left; *p* = 0.380; bilateral degeneration was observed in 14.8% (23/155) of patients. The inter-rater reliability for TMJ degeneration grading was 0.664 according to Cohen’s kappa, indicating substantial agreement.

### Discal collagen density

**Fig. 4:**
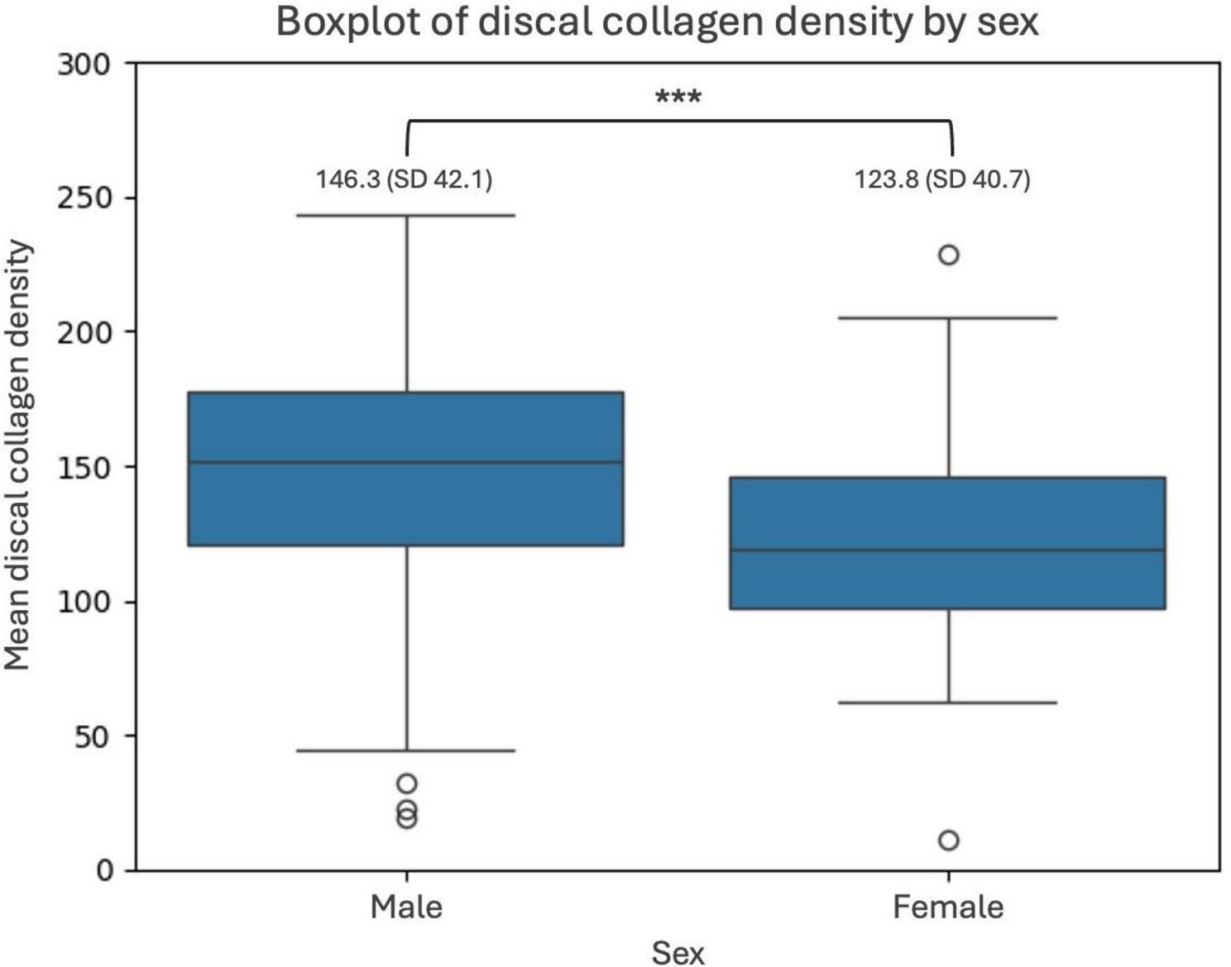
Boxplots of discal collagen density by sex. Boxplots shows unadjusted collagen density. After adjusting for TMJ degeneration, the mean discal collagen density was 146.3 (SD 42.1) in males and 123.8 (SD 40.7) in females (*p* < 0.001)

Unadjusted mean collagen density was 142.3 (SD 42.7; range 11.4–243.2). GEE in the whole cohort showed significant negative associations between discal collagen density and presence of TMJ degeneration (beta − 11.4, 95%CI -22.3, -0.6, *p* = 0.039) and female sex (beta − 22.8, 95%CI -36.7, -8.8, *p* = 0.001), meaning that exhibiting degeneration and female sex were both associated with lower collagen densities, independent of each other. In this analysis, neither age (beta 0.3, 95%CI -0.1, 0.7, *p* = 0.114) nor history of radiation- (beta − 7.0, 95%CI -25.9, 11.9, *p* = 0.469) or chemotherapies (beta − 0.2, 95%CI -20.6, 20.3, *p* = 0.987) were independently associated with discal collagen density. Thus, when adjusting for degeneration, mean discal collagen density was 123.8 (SD 40.7) in females and 146.4 (SD 42.1) in males (*p* < 0.001) – a boxplot of collagen densities, with adjusted means is given as Fig. 4.

## Discussion

This study is the first to report collagen density measurements of the discal space in the TMJ using DECT. We found that females and individuals with TMJ degeneration exhibit significantly lower collagen density compared to males. While this aligns with prior research suggesting that estrogen influences collagen metabolism, potentially leading to reduced collagen density and increased susceptibility to degeneration in females, it is important to note that the role of collagen changes in TMJ degeneration requires further investigation. Structural degradation of the joint is indeed associated with a progressive loss of collagen integrity, but the exact relationship between these factors needs to be explored in more detail.

Quantification of collagen density in the disc provides a valuable foundation for understanding the structural and functional integrity of the TMJ. Several studies have explored the relationship between collagen density and degenerative joint in other joints. A study on mouse osteoarthritis demonstrated structural changes in collagen networks across joint tissues, including cartilage and subchondral bone, highlighting collagen fiber thickening and dysregulation as key features of degeneration [18]. The results of our analysis could serve as a starting point for future quantitative studies, particularly in conjunction with magnetic resonance imaging (MRI) using T1rho or ultra-short time to echo (UTE) [19] sequences – this combination may generate a more comprehensive assessment of both morphological and biochemical changes in the TMJ. Recent advances in MRI-based quantitative imaging further emphasize the relevance of such multimodal approaches. Texture analysis of MRI data can capture subtle disc alterations associated with joint effusion, thereby providing quantitative insights into disc composition and degeneration [20]. Moreover, MRI-based radiomics has been applied for the evaluation of TMJ disc displacement [21], highlighting the potential of advanced imaging features to improve diagnostic accuracy beyond conventional imaging techniques. Importantly, collagen density mapping using DECT offers a complementary approach to MRI: it allows for high-resolution, three-dimensional assessment of the disc’s collagen content and structural integrity, which may detect subtle degenerative changes before overt morphological changes become visible on MRI. In addition, DECT collagen density mapping can be performed during a routine CT examination, providing a faster and more accessible assessment compared with MRI, which is often time-consuming, less widely available, and subject to contraindications such as claustrophobia or implanted devices. This relative advantage may broaden its clinical applicability, particularly in oncologic patients who frequently undergo CT staging as part of routine care. Given the potential of spectral CT in evaluating joint integrity, this method may be particularly useful in conditions such as early-stage osteoarthritis, where subtle changes in collagen integrity could serve as an early marker of disease progression. In trauma-related disc pathology, collagen density mapping may aid in assessing structural damage and contribute to preoperative planning by providing a precise evaluation of disc composition and degeneration. Additionally, photon-counting CT’s higher resolution and spectral capacities could offer improved tissue characterization [22], which may enhance early detection and monitoring in clinical practice.

Previous studies showed sex-specific differences in the prevalence and clinical features of TMJ disorders [23]. TMJ disorders, particularly temporomandibular dysfunction, occur more frequently in females than in males [24]. Reasons for this disparity are multifactorial, including factors such as sex-specific differences in the TMJ anatomy with a smaller joint surface area in females. Additionally, hormonal influences are being discussed [25]. Studies have reported that a majority of patients presenting with TMJ symptoms are between 20 and 50 years of age [24, 26]. Furthermore, various tissues exhibit decreased collagen density with advancing age [27], and these changes in composition may also be associated with changes in the organizational structure of the collagen matrix. Within the current study, age was not found to generate a significant effect independent of degeneration and sex– this may be explained by our cohort’s mean age being skewed towards older individuals.

Some limitations need to be discussed. The most important limitation of this study is the absence of a histopathological reference standard, which would be required to directly validate collagen density measurements. Moreover, the study cohort consisted of oncological patients without a specific TMJ disease background, which may limit the generalizability of our findings to patients with primary TMJ disorders. Our analysis was based solely on CT imaging techniques without additional clinical information, such as known TMJ disorders, TMJ pain, patients’ body mass index, or systemic connective tissue disorders, which could not be considered as potential confounders. Furthermore, we were unable to assess the influence of prior treatments on collagen density, as detailed radiotherapy plans and information on specific chemotherapeutic agents were not available for the included patients. The disc was not directly visualized, which may have led to potential inaccuracies in the measurements. Therefore, using a fixed 8 mm^2^ oval ROI, there is a possibility that some regions of interests may have included tendons or other tissues that also play a key role in understanding TMJ degeneration. A more precise differentiation between these tissues may be necessary to make more accurate statements regarding collagen density in the disc. Furthermore, inter- and intra-observer variability of ROI placement was not assessed. The second exam was excluded to ensure consistency. Evaluating measurement stability between exams could provide insights into reproducibility for future research. Future studies should validate DECT-derived collagen density against MRI biomarkers (e.g., T1rho, UTE) and, where feasible, histopathology, and include reproducibility assessments with multiple readers to further evaluate the robustness of the findings. Due to sex-based disparities in the likelihood of malignancies of the neck [28], our study cohort is predominantly male. Consequently, our results may not be fully generalizable to other patient groups as this discrepancy could influence the interpretation of the findings. Future studies should aim for a balanced sex-ratio to ensure that insights can be applied to a broader population.

## Conclusions

The possibility of complementing the clinical standard procedure of MRI with DECT opens new perspectives for the understanding of tissue changes underlying both natural aging and pathological changes of the TMJ. DECT provides both more detailed structural information regarding bony changes and compositional data on the collagen density of the disc. These insights could contribute to developing a more comprehensive understanding of the pathological processes in the TMJ, thereby improving treatment options.

## Data Availability

The data presented in this study are available on reasonable request from the corresponding author.
